# Quantitative Analysis of Abdominal Muscles Using Elastography in Female Patients With Incisional Hernia

**DOI:** 10.3389/fsurg.2022.831184

**Published:** 2022-04-12

**Authors:** Bo Wang, Kai He, Yulan Zhu, Xiaojian Fu, Qiyuan Yao, Hao Chen, Xiaohong Wang

**Affiliations:** ^1^Department of Ultrasound, Huashan Hospital, Fudan University, Shanghai, China; ^2^Department of General Surgery, Huashan Hospital, Fudan University, Shanghai, China; ^3^Department of Rehabilitation, Huashan Hospital, Fudan University, Shanghai, China

**Keywords:** abdominal wall muscle, incisional hernia, shear wave elastography, virtual touch tissue imaging quantification, biomechanical property

## Abstract

This study aimed to assess the thickness and shear wave speed (SWS) of the anterolateral abdominal wall muscles in female patients with incisional hernias of different widths, in order to analyze the biomechanical properties of abdominal wall muscles. This study included 53 patients with incisional hernia (Group A [hernia width <4 cm]: 21 patients, Group B [hernia width ≥4 cm]: 32 patients). The muscle thickness and SWS values of the external oblique (EO), internal oblique (IO), and transversus abdominis (TrA), and the hernia width were measured using Siemens Acuson S2000 ultrasound systems. Four detection points were labeled on the anterolateral abdominal wall: points 1, 2, 3, and 4, corresponding to the upper right, upper left, lower right, and lower left, respectively. The muscle thickness of the IO at point 3 was significantly different between both groups (*p* = 0.024). Group B had significantly higher SWS values than Group A, especially for the EO (points 1, 2, and 3), IO (points 1 and 2), and TrA (points 2 and 4) (*p* < 0.05). Pearson correlation analysis shows no significant correlation between muscle thickness and the SWS values of EO, IO, and TrA (all *p* > 0.05). Linear correlation analysis showed a significantly positive correlation between hernia width and the mean SWS value of EO, IO, and TrA (*p* = 0.004, 0.005, and 0.043, respectively). Muscle thickness was not reliable measure to directly reflect the biomechanical changes of the abdominal wall muscles in patients with incisional hernia. Comparatively, SWE can accurately measure the stiffness of the abdominal wall muscles and intuitively evaluate its biomechanical properties.

## Introduction

Incisional hernia, a common complication of abdominal surgery, is reported to have an incidence of around 11–20% (1). A previous study reported that the 10-year cumulative rate of recurrence for suture and prosthetic repair was 63 and 32%, respectively (2). Additionally, failed surgical repair and hernia recurrence predispose patients to further complications, as each subsequent repair imposes greater technical challenges, thus initiating a vicious cycle (3).

A recent study demonstrated that an increase in hernia width was significantly associated with recurrence (4), and that failed surgical repair may be caused by severe lateral retraction and tension of the abdominal muscles. Recently, botulinum toxin injections have been identified as a potential pre-operative means to counteract the abdominal wall tension, reduce hernia size, and facilitate fascial closure during hernia repair. Moreover, the curative effect of injection is generally seen while increasing the length and decreasing the thickness of the lateral abdominal muscles (3). Although botulinum (Botox) injections in the anterolateral muscles of the abdominal wall reduce the difficulty of repairing large incisional hernias, there remains a lack of technical standardization, such as the identification of optimal injection sites, volume, and concentration, as well as a proper description of its safety profile, among others. Thus, a real-time, noninvasive, and quantitative imaging tool is desired to evaluate the biomechanical properties of the anterolateral abdominal wall musculature before and after Botox injections.

Shear wave elastography (SWE) can be used to obtain quantitative biomechanical information regarding the tissue through noninvasive examination, which can provide important and objective information for clinical use. A linear relationship between the measured shear elastic modulus (or shear wave speed) and muscular tension has been reported (5, 6). In recent years, the application of SWE in limb muscles has grown exponentially (7–9) but studies regarding its application on abdominal wall muscles are still lacking. Recently, a study on patients with incisional hernia showed that SWE based on virtual touch tissue imaging quantification (VTIQ) can assess the elastic properties of abdominal muscles in real time with excellent reproducibility in terms of intra- and inter-operator reliability of the external oblique (EO) and internal oblique (IO). Furthermore, the shear wave speed (SWS) values in incisional hernia patients were higher than those in healthy subjects (10).

Because many unknowns are yet to be addressed by studies of the human abdominal wall, more data on the biomechanical properties of the abdominal wall is needed before the development of guidelines for hernia repair and the prevention of recurrence, including elasticity indicators. This study aims to analyze the SWS of the anterolateral abdominal wall muscles, including the EO, IO, and transversus abdominis (TrA) in patients with incisional hernias of different widths.

## Materials and Methods

### Participants

Approval for the research was received from the Institute Ethics Committee of Huashan Hospital (No.KY2018-438). Informed consent forms were signed by all participants. The study protocol was registered in China Clinical Registry Center (ChiCTR1900023012).

**Figure 1:**
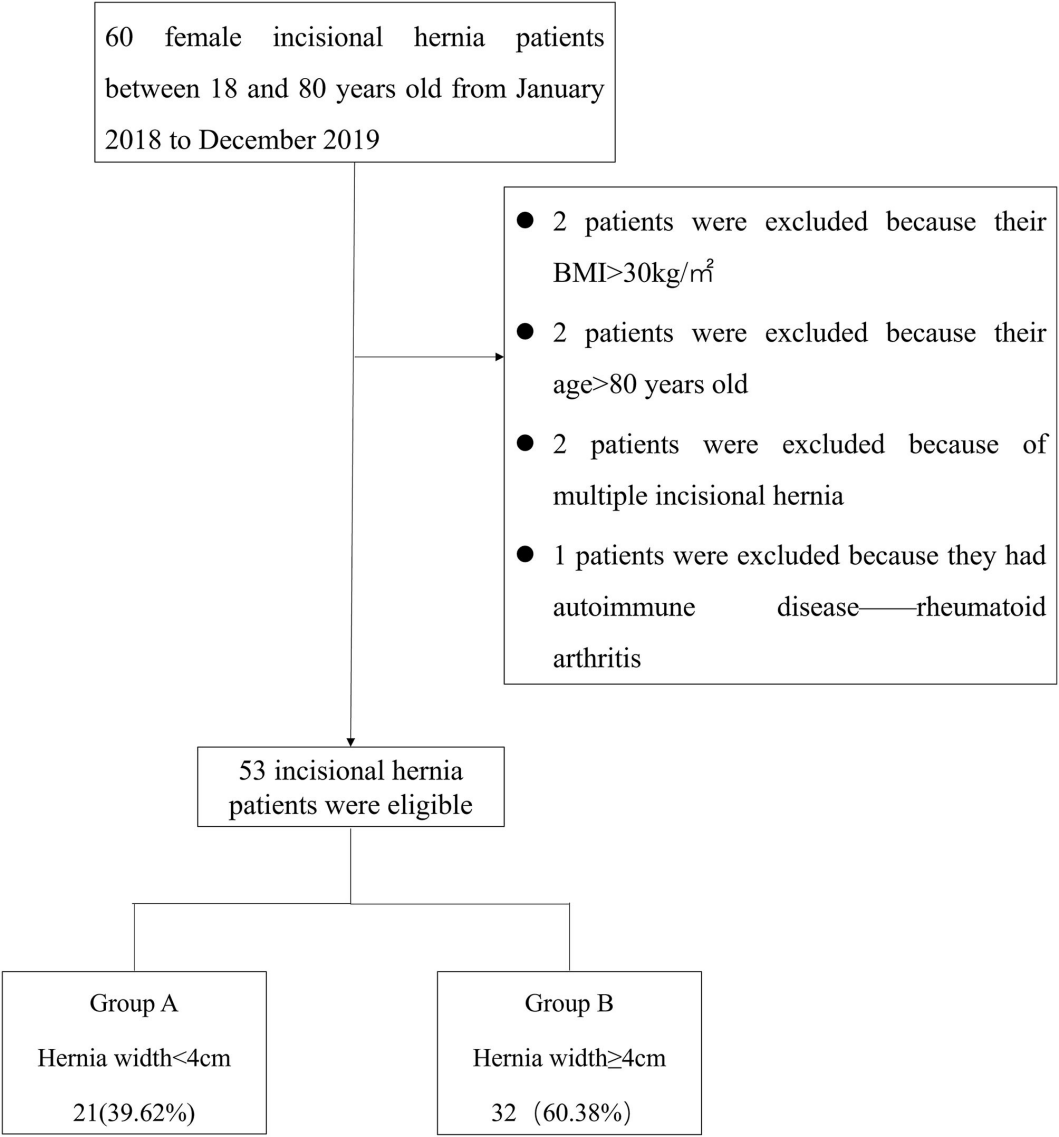
Schematic for the classification of incisional hernia patients by hernia width.

Retrospective analysis of prospectively collected data on results and consequences of 60 patients diagnosed with incisional hernia at our institution underwent ultrasound and SWE between January 2018 and December 2019. The inclusion criteria were as follows: (1) female, (2) 18–80 years old, and (3) body mass index (BMI) <30 kg/m^2^. The exclusion criteria were as follows: (1) having chronic or degenerative pathology of the muscle (e.g., autoimmune myositis), (2) American Society of Anesthesiologists score ≥3, (3) receiving myorelaxants, corticosteroids, or immunosuppressive medications at the time of the study, (4) participation in professional athletic sports or exercise within 48 h prior to the test, and (5) having multiple incisional hernias. Patients' age, BMI, incisional hernia location, and duration of incisional hernia were recorded. Of these patients, 7 were excluded due to meeting the exclusion criteria of BMI >30 kg/m^2^ (*n* = 2), Age > 80 years old (*n* = 2), having multiple incisional hernias (*n* = 2), or having autoimmune disease (rheumatoid arthritis; *n* = 1). Finally, 53 patients were included in this study (Figure 1).

Hernia width was defined as the maximum horizontal distance between the two lateral edges of the hernia. The European Hernia Society classification divides incisional hernias into 3 subclasses: W1 <4 cm (small), 4 ≤ W2 <10 cm (medium), and W3 ≥ 10 cm (large) (11). Small incisional hernias were included in Group A, while medium and large hernias were included in Group B. Figure 1 shows the distribution of the study patients in each group.

### Equipment

Ultrasound images and SWS measurements of the three anterolateral abdominal walls (EO, IO, TrA) were obtained using the Siemens Acuson S2000 ultrasound systems (Siemens Medical Inc., Mountain View, CA, USA), equipped with a linear array transducer (9L4) ranging from 4 to 9 MHz in frequency. The SWS can be converted to the shear elastic modulus using the formula G = ρc^2^, wherein G is the shear elastic modulus, ρ is the density, and c is the shear wave propagation speed. However, it is important to consider that this calculation assumes an isotropic tissue with uniform density. For this reason, quantitative shear wave measurements in the musculoskeletal system are often reported as speed (m/s), rather than tissue elasticity (kPa).

### Study Design

**Figure 2:**
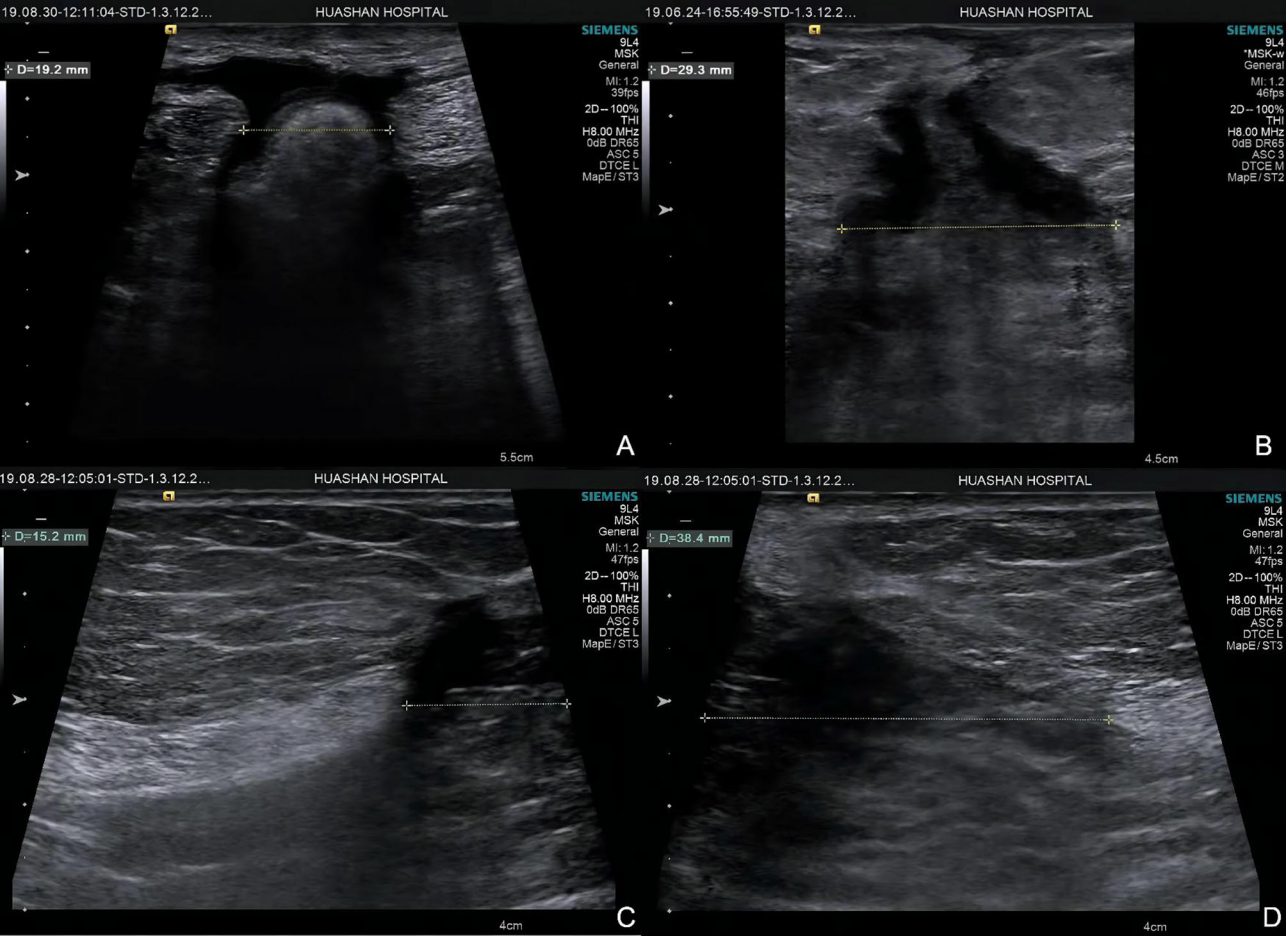
Hernia width of two groups. Group A: **(A)** hernia width = 19.2 mm; **(B)** hernia width = 29.3 mm. Group B **(C,D)**: hernia width = 15.2 + 38.4 = 53.6 mm.

**Figure 3:**
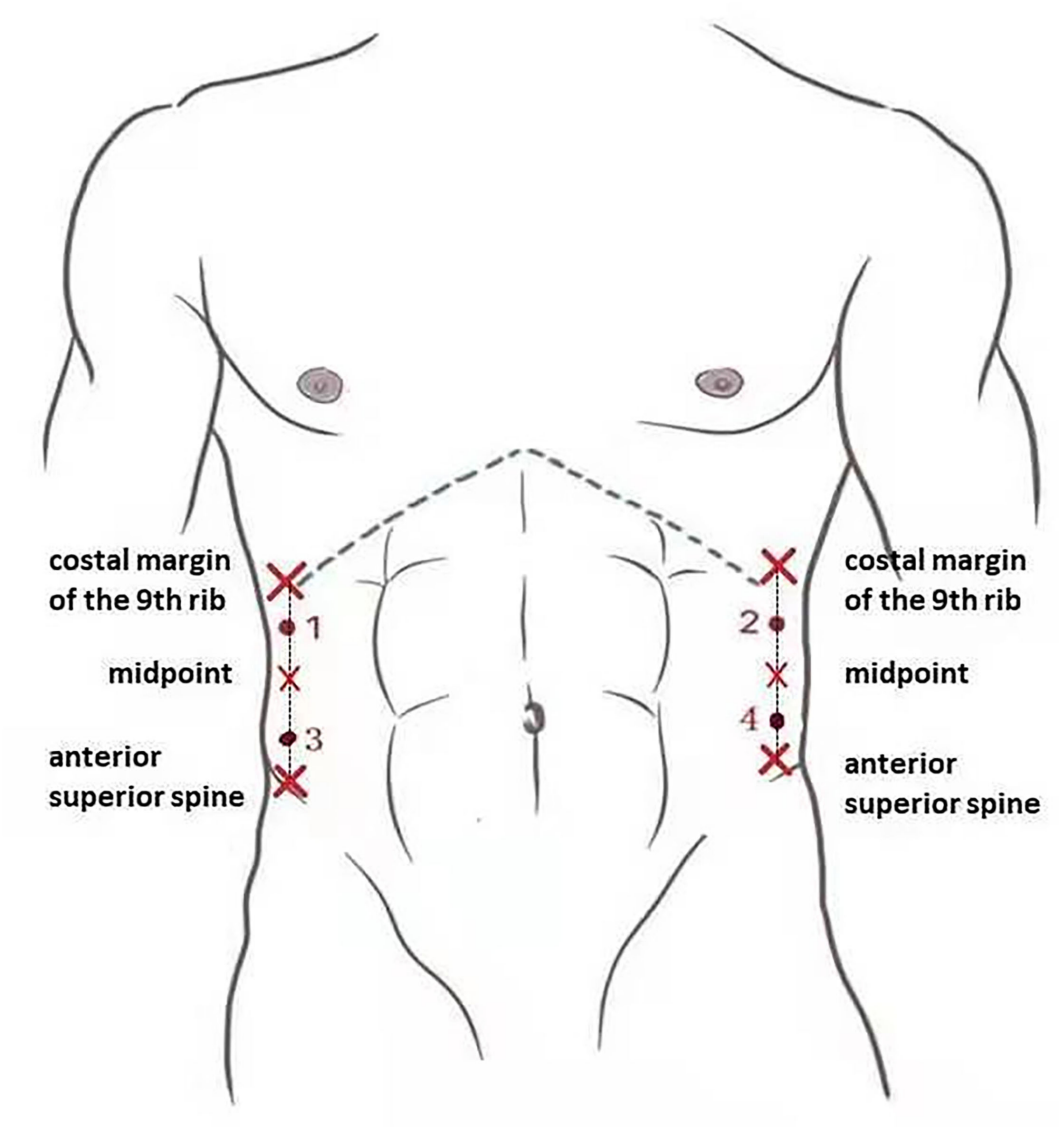
The four measurement points used for shear wave elastography on the anterolateral abdominal wall muscles.

**Figure 4:**
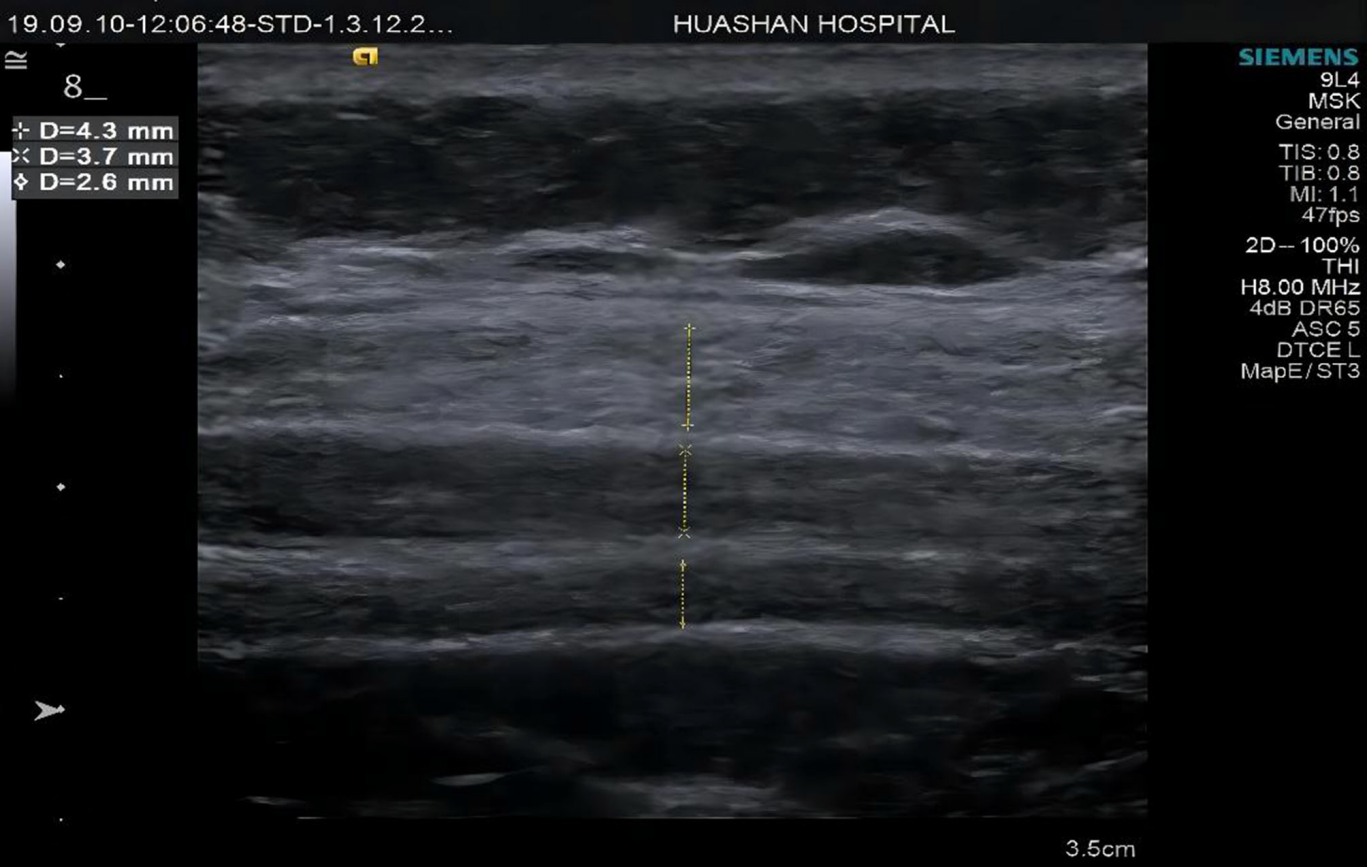
Muscle thickness measurements diagrams of Group A at point 2 external oblique muscle (EO), internal oblique muscle (IO), and transverse abdominis (TrA).

All measurements were performed by 2 sonographers with 10 and 4 years of experience in elastography, respectively. Subjects were asked to lie in the supine position and instructed to breathe normally and relax their abdomen. The width of the incisional hernia was obtained by measuring the myofascial length between the left and right sides in the grayscale B-mode US by the same sonographer (Figure 2). Along the anterior axillary line, the midpoint of the line between the costal margin of the ninth rib and the anterior superior iliac spine was taken to divide the abdomen into upper and lower halves. Two points on each side of the abdomen would be identified at the midpoint of the upper and lower halves, according to the injection sites of botulinum identified by Elstner et al. (12). These points were labeled as point 1, point 2, point 3, and point 4, corresponding to the upper right, upper left, lower right, and lower left points, respectively (Figure 3). The ultrasound transducer was placed in the transverse orientation and perpendicular to the skin with the least amount of pressure possible. A thick layer of acoustic paste was applied to block the air and cushion the pressure of the probe. The muscle thickness was obtained by measuring the distance between the superficial and deep myofascial layers (Figure 4).

Afterwards, we switched the machine to the “VTIQ” mode and adjusted the elastography box to the appropriate size (width adjusted to the maximum depth of the deep surface of TrA). The quality score was interpreted using a red-yellow-green color scheme representing low to high quality with a uniform green image representing the highest quality.

**Figure 5:**
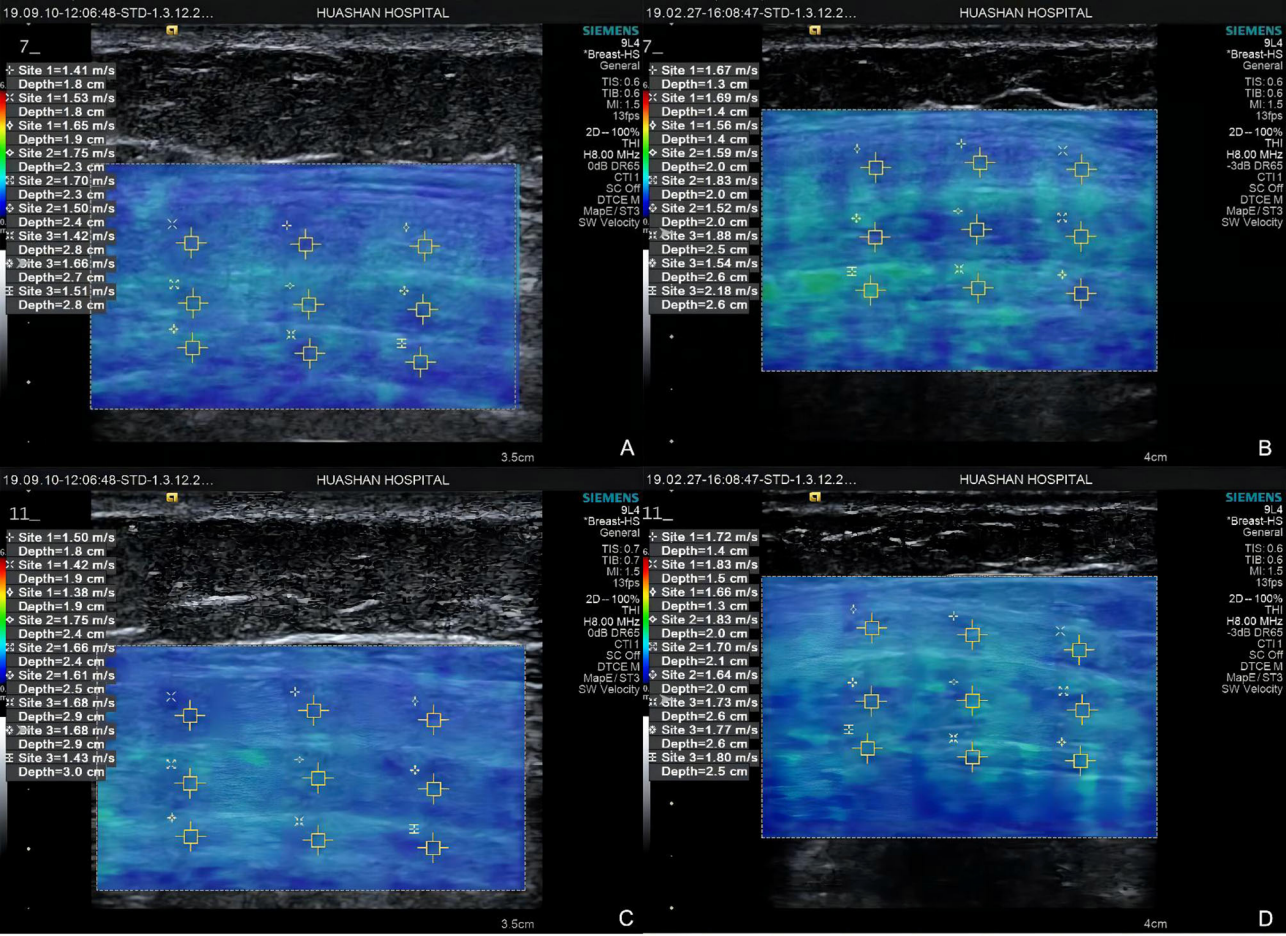
Elastograms at point 1 of Group A **(A)** external oblique muscle, internal oblique muscle, and transverse abdominis) and Group B **(B)** external oblique muscle, internal oblique muscle, and transverse abdominis), point 3 of Group A **(C)** external oblique muscle, internal oblique muscle, and transverse abdominis) and Group B **(D)** external oblique muscle, internal oblique muscle, and transverse abdominis).

Regarding the speed pattern, the values of SWS were superimposed on a two-dimensional gray scale image with color coding (red for high, blue for low, and yellow or green for intermediate), and the maximum velocity was set at 6.5 m/s. Afterwards, 3 small square ROIs (a system tool used to automatically quantify the SWS) with a diameter of 1.5 mm were positioned at 3 points (central, left, and right) quartering the muscle on the elastogram, and their sizes were set to the minimum TrA thickness suitable for all participants (Figure 5). The ROIs were placed on the muscle belly, avoiding the tendons, aponeurosis, blood vessels, and fascial tissues. A higher speed value represents a harder tissue property. The mean value of the three points represented the final measurement of the muscle. For each patient, measurements were taken three times for each point, and three elastography images were acquired in each region. The mean measurement of SWS was subsequently recorded.

### Statistical Analysis

The SPSS 22.0 software was used for statistical analysis. Continuous variables were expressed as their mean ± standard deviation (SD). The intra- and inter-operator reliability was assessed using intra-class correlation coefficients (ICC). The ICC was interpreted as either excellent (1.00–0.75), moderate (0.74–0.60), fair (0.59–0.40), and poor (<0.40) (13). Independent sample *t*-tests involving 2 groups were used to compare values between the groups. Pearson correlation analysis was used to evaluate the correlation between muscle thickness and SWS. The correlation between hernia width and SWS value was examined using linear regression analysis. Two-tailed *p*-values of < 0.05 were considered statistically significant.

## Results

### Basic Characteristics

A total of 53 patients with incisional hernia (age: 64.9 ± 8.1 [45–79] years; BMI: 24.11 ± 2.68 [17.7–28.5] kg/m^2^; duration of incisional hernia: 13.7 ± 22.4 [1–120] months; range, months) were included in the study. Group A had 21 patients with a mean hernia width of 2.82 ± 0.79 (1.03–3.88) cm, whereas Group B had 32 patients with a mean hernia width of 5.80 ± 1.65 (4.18–11.24) cm (Table 1).

**Table 1:** Basic characteristics of group A and group B.

		**Group A (*n =* 21)**	**Group B (*n =* 32)**	***P***
Age(years)	Min	49	45	0.629
	Max	77	79	
	Mean ± SD	65.60 ± 8.00	64.47 ± 8.25	
BMI (kg/m^2^)	Min	17.7	19.2	0.691
	Max	27.3	28.5	
	Mean ± SD	23.92 ± 2.91	24.23 ± 2.56	
Duration of incisional hernia(months)	Min	1	1	0.138
	Max	120	48	
	Mean ± SD	21.32 ± 33.48	9.13 ± 10.04	
Hernia width (cm)	Min	1.03	4.18	0.000
	Max	3.88	11.24	
	Mean ± SD	2.82 ± 0.79	5.80 ± 1.65	

*Min, minimum; Max, maximum; Mean ± SD, mean ± standard deviation; BMI, body mass index*.

### Comparison of Muscle Thickness at Four Points

No statistical differences were noted for age, BMI, and duration of incisional hernia between the two groups (*p* > 0.05). As shown in Table 2, the anterolateral abdominal wall muscle thickness of Group B was higher than that of Group A, but the difference was statistically significant only for the IO at point 3 (7.78 ± 1.77 vs. 6.73 ± 1.28, *p* = 0.024).

**Table 2:** Comparison of muscle thickness between group A and group B.

	**Thickness (mm)**		***T***	**Mean difference, 95%CI**	***P***
	**Group A (*n =* 21)**	**Group B (*n =* 32)**			
**EO**
Point 1	4.12 ± 0.96	4.24 ± 0.63	−0.508	−0.12 [−0.60, 0.36]	0.615
Point 2	3.97 ± 1.03	4.18 ± 0.95	−0.738	−0.20 [−0.76, 0.35]	0.464
Point 3	4.38 ± 0.89	4.70 ± 0.99	−1.198	−0.32 [−0.85, 0.22]	0.237
Point 4	4.04 ± 0.77	4.38 ± 0.92	−1.388	−0.34 [−0.82, 0.15]	0.171
**IO**
Point 1	4.98 ± 1.17	5.59 ± 1.66	−1.464	−0.61 [−1.45, 0.23]	0.149
Point 2	5.14 ± 1.51	5.30 ± 1.19	−0.432	−0.16 [−0.91, 0.58]	0.668
Point 3	6.73 ± 1.28	7.78 ± 1.77	−2.331	−1.05 [−1.95, −0.15]	0.024
Point 4	7.11 ± 1.54	7.57 ± 1.56	−1.034	−0.45 [−1.33, 0.43]	0.306
**TrA**
Point 1	2.68 ± 0.58	2.93 ± 0.63	−1.452	−0.25 [−0.59, 0.10]	0.153
Point 2	2.51 ± 0.48	2.81 ± 0.75	−1.625	−0.30 [−0.67, 0.07]	0.110
Point 3	2.50 ± 0.61	2.58 ± 0.71	−0.416	−0.08 [−0.45, 0.30]	0.697
Point 4	2.41 ± 0.56	2.64 ± 0.69	−1.254	−0.23 [−0.59, 0.14]	0.216

*EO, external oblique muscle; IO, internal oblique muscle; TrA, transversus abdominis*.

### Reliability

We performed a reliability analysis of the SWS on the anterolateral abdominal muscles at all four points, and both the overall intra- and inter-operator reliability were excellent (ICC = 0.86 and 0.82, respectively). The inter-operator agreement of EO, IO, and TrA was slightly lower than the intra-operator reproducibility (ICC range: 0.76–0.81 and 0.81–0.86, respectively; Table 3).

**Table 3:** Reliability of SWS measured in the three abdominal muscle.

	**EO (*n =* 212)**	**IO (*n =* 212)**	**TA (*n =* 212)**
**Intra-operator reliability**
ICC	0.81	0.86	0.84
95%CI	(0.76–0.85)	(0.82–0.89)	(0.80–0.88)
**Inter–operator reliability**
ICC	0.76	0.79	0.81
95%CI	(0.70–0.81)	(0.73–0.83)	(0.75–0.85)

*EO, external oblique muscle; IO, internal oblique muscle; TrA, transversus abdominis; ICC, intraclass correlation coeffificient*.

### Comparison of SWS at Four Points

As shown in Table 4, there were statistically significant differences in SWS values at four points between the two groups, especially for the EO (points 1, 2, and 3), IO (points 1 and 2), and TrA (points 2 and 4) (*p* <0.05). In general, Group B had significantly higher SWS values than Group A (1.71 ± 0.25 vs. 1.55 ± 0.13, respectively; *p* = 0.004).

**Table 4:** Comparison of shear wave speed between group A and group B.

	**SWS (m/s)**		***T***	**Mean difference, 95%CI**	***P***
	**group A (*n =* 21)**	**group B (*n =* 32)**			
**EO**
Point 1	1.34 ± 0.15	1.46 ± 0.24	−2.149	−0.13 [−0.24, −0.01]	0.036
Point 2	1.41 ± 0.18	1.55 ± 0.27	−2.044	−0.14 [−0.27, −0.00]	0.046
Point 3	1.42 ± 0.16	1.57 ± 0.23	−2.602	−0.15 [−0.27, −0.03]	0.012
Point 4	1.47 ± 0.17	1.56 ± 0.26	−1.429	−0.09 [−0.22, 0.04]	0.159
**IO**
Point 1	1.54 ± 0.18	1.70 ± 0.31	−2.199	−0.17 [−0.32, −0.01]	0.032
Point 2	1.56 ± 0.17	1.76 ± 0.29	−3.257	−0.21 [−0.33, −0.08]	0.002
Point 3	1.56 ± 0.16	1.67 ± 0.26	−1.785	−0.11 [−0.24, −0.01]	0.080
Point 4	1.58 ± 0.22	1.71 ± 0.29	−1.756	−0.13 [−0.28, −0.02]	0.085
**TrA**
Point 1	1.67 ± 0.18	1.83 ± 0.41	−1.963	−0.16 [−0.33, −0.00]	0.056
Point 2	1.68 ± 0.17	1.88 ± 0.36	−2.634	−0.19 [−0.34, −0.05]	0.011
Point 3	1.68 ± 0.19	1.81 ± 0.37	−1.694	−0.13 [−0.29, 0.02]	0.097
Point 4	1.66 ± 0.25	1.86 ± 0.32	−2.340	−0.19 [−0.36, −0.03]	0.023

*EO, external oblique muscle; IO, internal oblique muscle; TrA, transversus abdominis; SWS, shear wave speed*.

### Correlation Between Muscle Thickness and SWS

No significant correlation was noted between the mean SWS of EO (*r* = −0.178, *p* = 0.202), IO (*r* = 0.043, *p* = 0.765), and TrA(r = 0.147, *p* = 0.293) and the mean muscle thickness. Similarly, there was no correlation between the SWS of the four points of EO, IO, and TrA and their corresponding muscle thicknesses (all *p* > 0.05).

### Correlation Between Hernia Width and the Mean SWS

**Figure 6:**
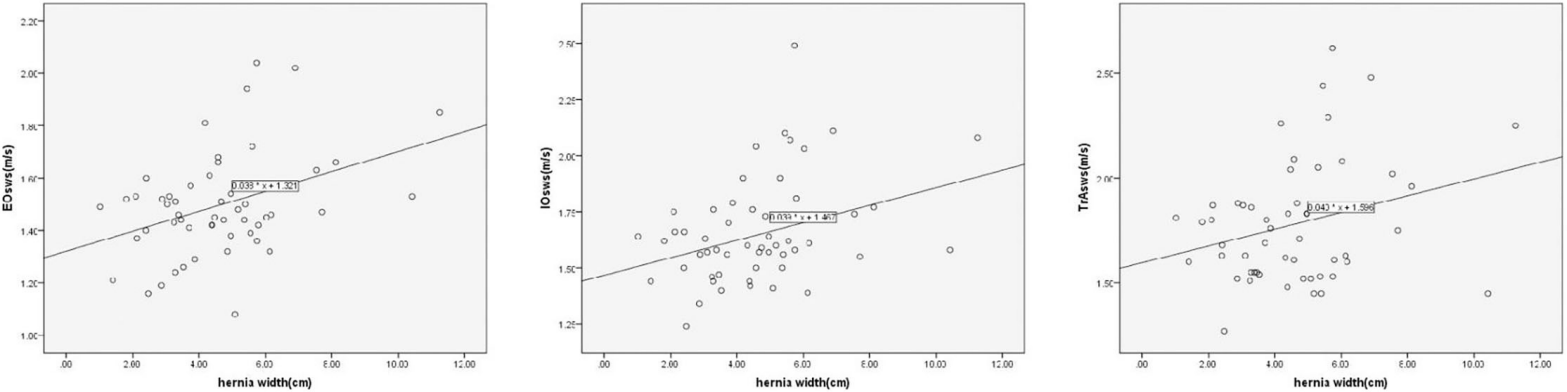
Linear correlation analysis shows significantly positive correlation between hernia width and the mean SWS value of the EO, IO, and TrA (*p* = 0.004, 0.005, and 0.043, respectively).

On linear regression analysis, the regression equation for EO was Y = 1.321 + 0.038X, using hernia width as the independent variable and the mean SWS value of EO as the dependent variable, with a significant correlation coefficient of *R* = 0.386, *R*^2^ = 0.149, and adjusted *R*^2^ = 0.132 (*F* = 8.904, *p* = 0.004). Likewise, the regression equation for IO was Y = 1.467 + 0.039X, with a significant correlation coefficient of *R* = 0.380, *R*^2^ = 0.145, and adjusted *R*^2^ = 0.128 (*F* = 8.460, *p* = 0.005). Similarly, there was a significant positive correlation between hernia width and the mean SWS value of TrA. The regression equation was Y = 1.596 + 0.040X, with a significant correlation coefficient of *R* = 0.279, *R*^2^ = 0.078, and adjusted *R*^2^ = 0.060 (*F* = 4.315, *p* = 0.043) (Figure 6). To ensure the representativeness of the data, outlier data of the IO (2.49 m/s for SWS) was eliminated.

## Discussion

Since one objective of hernia treatment is to restore the mechanical function of the abdominal wall, this would benefit from a better understanding of the mechanical behavior of the abdominal wall. However, previous approaches to the mechanics of the abdominal wall are invasive, and when an identified tissue is isolated from the whole, the loss of mechanical resistance may alter the mechanical response of the tissue (14). On the other hand, SWE enables the assessment of muscle elasticity through a non-invasive estimation of the apparent shear modulus *in situ*, and the resulting quantitative parameters of elasticity could be used to establish or validate a finite element model of the abdomen (15).

Due to its novelty, different articles studying the use of SWE on abdominal muscles have various research designs and methodologies. In addition to the inherent challenges of muscular anisotropy, the reliability of abdominal muscle elastography still remains controversial because of the abdominal muscles perform different functions compared to the limbs. Furthermore, the abdominal muscles are affected by breathing movements at rest. Therefore, the primary purpose of this study was to explore the feasibility of the application of SWE for the anterolateral abdominal wall muscles, as well as to design a method for assessing the differences of the biomechanical properties of anterolateral abdominal wall muscles in patients with incisional hernias of different widths.

### Biomechanical Information Provided by Changes in Muscle Thickness

After its formation, the fascial separation and loss of midline muscle attachment in large ventral hernias lead to abdominal muscle shortening and relative unloading, particularly in the lateral oblique muscles which has lost its insertions (16, 17). Other models of muscle unloading demonstrate that muscle atrophy with a reduction in fiber diameter begins within 3–5 days. The muscles of the abdominal wall are composed primarily of slow twitch fibers. Slow twitch predominant muscles are more susceptible to disuse atrophy than fast twitch muscles (18). In line with this, Wang et al., reported that the muscle thickness of the IO and TrA was significantly thinner in those with incisional hernia vs. healthy subjects (*p* = 0.011 and 0.047, respectively).

In our specific 4-point measurement, we observed that Group B has greater muscle thickness than Group A, but the difference was not statistically significant except for point 3 of the IO. This suggests that larger hernia width may reflect the more obvious contracture of the muscle. However, in addition to contracture, changes in muscle fiber diameter and muscle atrophy occur after hernia formation; and thus, muscle thickness cannot directly reflect the biomechanical changes of the abdominal wall muscle. This study also demonstrated that muscle thickness and stiffness were not correlated.

### Reliability of SWS Measurements in Anterolateral Abdominal Wall Muscles

In our study, the reliability of elastography measurements was excellent in all examined muscles (ICC range: 0.76–0.86), which was somewhat similar to the results of other studies (19). However, compared to the results of MacDonald et al. (20), the reliability of the transverse abdominis muscle of our study was similar for the superficial muscles (EO and IO) (ICC: 0.81, 0.86, and 0.84 vs. ICC: 0.89, 0.61, and 0.45, for the EO, IO, and TrA, respectively). There are several possible reasons for this finding. First, the degree of the proficiency in the application of this technique may have affected the level of reliability. Our study was conducted by two skilled operators who have been using VTIQ for more than 2 years. Hirayama et al. (21) described the possibility of this hypothesis in their study. Second, the differences in equipment with the other two studies that used another scanner (Aixplorer, Supersonic Imagine, France) that calculated in kilopascals may have affected their results. Our results were calculated in meters per second on an Acuson S2000 scanner. Third, the 4-point measurement method we applied enabled us to obtain higher values than the 2-point measurement method utilized in previous literature because of the possibility of joint decision-making. Fourth, in theory, patients with high BMI may have more variability in the measurement of abdominal wall muscle SWS due to thicker subcutaneous fat (22). Since the BMI of all the patients included in our study was <30 kg/m^2^, with an average of 24.11 ± 2.68 kg/m^2^, the accuracy and stability of SWS detection may be better, suggesting higher reliability.

Gabrielsen et al. (22) reported that the SWS in healthy people ranged from 1.40 to 2.96 m/s, similar to our findings (range: 0.95–2.83 m/s). MacDonald et al. (20) evaluated the shear modulus of EO, IO, and TrA in young healthy volunteers at rest, with corresponding mean values ranging from 3.5 to 7.2 kPa, which is consistent with our findings through formula conversion.

### Effect of Hernia Width on Abdominal Muscles Stiffness

A previous study found that the integrity and tension balance of the abdominal wall are disrupted following the formation of incisional hernia, and the abdominal wall length decreased by 20% in these patients vs. healthy controls on each side without the excision of any abdominal muscle (16). This means that hernia defects do not enlarge simply because repetitive evisceration of peritoneal contents causes dilation of a fascial defect. Rather, the lateral muscular components of the abdominal wall retract away from the fascial defect, which may be a direct manifestation of myofibril atrophic changes. The number of sarcomeres arranged in series is known to decrease in chronically shortened muscles and manifests as decreased muscular compliance (16, 23). In our specific 4-point measurement, we observed that the muscles of Group B significantly exhibited greater stiffness than those in Group A, and the stiffness of the abdominal muscles increases as the width of the hernia increases. However, this effect was relatively low in our study. For every 1-cm increase in hernia width, the SWS of the EO, IO, and TrA only increased by 0.038, 0.039, and 0.040 m/s, respectively. Since most of the patients included in this study were elderly and only a few were under 60 years old, age stratification analysis was not conducted.

### Biomechanical Potential Properties in Herniated Abdominal Wall

Compared to the healthy subjects reported by Wang et al. (10), incisional hernia patients had higher SWS values, with statistically significant differences (*p* < 0.05). Following incisional hernia formation, the oblique muscles undergo pathologic changes consistent with disuse muscular atrophy resulting in changes in fiber-type composition (a fiber-type shift from type I to type II occurs, particularly with an increase in type IIa fibers), decreased cross-sectional area, and pathologic fibrosis. A *t*-test comparing the total muscular collagen between incisional hernia patients and uninjured subjects revealed a significant difference as well (8.3 ± 0.37% vs. 10.8 ± 0.63%, *p* < 0.01) (16). Degenerative muscle changes and alterations to the intramuscular connective tissue, including increased and disordered collagen production, may account for the pathological mechanical properties observed in the herniated abdominal wall (18). These changes effectively reduce the compliance of the abdominal wall and increases its stiffness.

The present study had a number of limitations. First, only a small number of patients had a large incisional hernia (width ≥10 cm), and this sample size will continue to increase with long-term studies. At the same time, a larger sample size is needed to verify the influence of incisional hernia location on the change in abdominal muscle stiffness. Additionally, the ROI for VTIQ (Siemens) was fixed in size and shape, and the results may not be sufficient to represent the entire muscle. A final possible limitation involves the unestablished protocols regarding transducer orientation (15, 20, 22, 24). Further research is needed to facilitate the development of standardized imaging protocols and positions.

In summary, we believe that SWE can accurately measure the stiffness of the abdominal wall muscles and intuitively evaluate its biomechanical properties. Further systematic analysis of the elastic characteristics of the abdominal wall muscles in patients with incisional hernia may help shed light on the pathological changes of the disease in order to improve hernia treatment, especially regarding the indications of Botox injections.

## Data Availability Statement

The original contributions presented in the study are included in the article/supplementary material, further inquiries can be directed to the corresponding author/s.

## Ethics Statement

The studies involving human participants were reviewed and approved by the Institute Ethics Committee of Huashan Hospital (No.KY2018-438). The patients/participants provided their written informed consent to participate in this study.

## Author Contributions

BW contributed to conceptualization and methodology. KH contributed to methodology and writing of the draft. YZ contributed to data collection. XF and QY contributed to statistical analysis. HC and XW contributed to reviewing and editing of the draft. All authors have read and approved the manuscript.

## Conflict of Interest

The authors declare that the research was conducted in the absence of any commercial or financial relationships that could be construed as a potential conflict of interest.

## Publisher's Note

All claims expressed in this article are solely those of the authors and do not necessarily represent those of their affiliated organizations, or those of the publisher, the editors and the reviewers. Any product that may be evaluated in this article, or claim that may be made by its manufacturer, is not guaranteed or endorsed by the publisher.
